# Metagenome-assembled genomes indicate that antimicrobial resistance genes are highly prevalent among urban bacteria and multidrug and glycopeptide resistances are ubiquitous in most taxa

**DOI:** 10.3389/fmicb.2023.1037845

**Published:** 2023-01-25

**Authors:** Stefanía Magnúsdóttir, Joao Pedro Saraiva, Alexander Bartholomäus, Majid Soheili, Rodolfo Brizola Toscan, Junya Zhang, Ulisses Nunes da Rocha, CLUE-TERRA consortium

**Affiliations:** ^1^Department of Environmental Microbiology, Helmholtz Centre for Environmental Research-UFZ, Leipzig, Germany; ^2^GFZ German Research Centre for Geosciences, Section Geomicrobiology, Potsdam, Germany; ^3^Department of Isotope Biogeochemistry, Helmholtz Centre for Environmental Research-UFZ, Leipzig, Germany; ^4^Research Center for Eco-Environmental Sciences, Chinese Academy of Sciences, Beijing, China

**Keywords:** Antimicrobial resistance, urban, prevalence, metagenome-assembled genome, bacteria, virulence factor, plasmid

## Abstract

**Introduction:**

Every year, millions of deaths are associated with the increased spread of antimicrobial resistance genes (ARGs) in bacteria. With the increasing urbanization of the global population, the spread of ARGs in urban bacteria has become a more severe threat to human health.

**Methods:**

In this study, we used metagenome-assembled genomes (MAGs) recovered from 1,153 urban metagenomes in multiple urban locations to investigate the fate and occurrence of ARGs in urban bacteria. Additionally, we analyzed the occurrence of these ARGs on plasmids and estimated the virulence of the bacterial species.

**Results:**

Our results showed that multidrug and glycopeptide ARGs are ubiquitous among urban bacteria. Additionally, we analyzed the deterministic effects of phylogeny on the spread of these ARGs and found ARG classes that have a non-random distribution within the phylogeny of our recovered MAGs. However, few ARGs were found on plasmids and most of the recovered MAGs contained few virulence factors.

**Discussion:**

Our results suggest that the observed non-random spreads of ARGs are not due to the transfer of plasmids and that most of the bacteria observed in the study are unlikely to be virulent. Additional research is needed to evaluate whether the ubiquitous and widespread ARG classes will become entirely prevalent among urban bacteria and how they spread among phylogenetically distinct species.

## Introduction

Every year, millions of human deaths can be associated with the spread of antimicrobial resistance genes (ARGs) in microbes, and antibiotic resistance will become the leading cause of death worldwide within the next decade (United Nations, 2016). Every year, the proportion of the global population living in urban environments increases, and to this date, more than half of the world population lives in urban environments (United Nations, 2018). People living in urban environments have more common surface areas that can be viewed as hot spots for microbial spread (e.g., public transit infrastructures, supermarkets, and park benches). People touch these common areas, leave, and pick up microbes through those contacts.

Antimicrobial resistance is a natural defense mechanism in bacteria and the spread of ARGs among bacteria is a naturally occurring phenomenon observed even in pristine environments. ARGs have, for example, been found in bacteria isolated from 2,000-year-old glacial ice and water samples (Dancer et al., 1997) and in metagenomic samples from over 10,000-year-old sediments (Song et al., 2005). However, because of the everyday use of antibiotics in healthcare and food production (United Nations, 2016), antimicrobial resistance is spreading among the microbes in our environment at an alarming rate.

A recent study showed that most samples from urban environments have some ARG levels (Danko et al., 2021). However, these studies did not report ARG spread among urban bacterial taxa. In this study, we aimed to investigate the spread of different types and classes of ARGs in urban bacteria. We gathered publicly available metagenomic sequences from studies that collected samples from urban environments. We then recovered metagenome-assembled genomes (MAGs) from each sample and searched for the presence of known ARGs in these genomes. Current protein alignment methods fail to identify over a third of microbial proteins (Bileschi et al., 2022). We, therefore, opted to use DeepARG (Arango-Argoty et al., 2018) to identify ARGs in urban MAGs. DeepARG is a deep learning model specifically trained to identify ARGs belonging to over 30 different classes in bacterial genomes.

Our findings show that ARGs are present at all branches in the phylogenetic tree of our recovered MAGs, and several ARG classes have already spread throughout the entire phylogenetic tree. We also see many ARG classes that are prevalent among urban bacteria but have not yet fully spread throughout the phylogenetic tree. These ARG classes need further investigation to evaluate how quickly we must act before these ARGs spread across the entire phylogenetic tree.

## Materials and methods

### Urban metagenome selection

We extracted metagenomic samples belonging to urban environments (TMDB biome: “urban”) from the Terrestrial Metagenome Database (Corrêa et al., 2019), which is a part of the Collaborative Multi-domain Exploration of Terrestrial metagenomes (CLUE-TERRA) consortium.^1^ Metagenomic samples in the CLUE-TERRA consortium have previously been filtered based on the following criteria: (i) Because non-metagenomic libraries in the Sequence Read Archive (SRA) can be wrongfully annotated as metagenomic, only true whole genome shotgun (WGS) libraries were kept. This was achieved using PARTIE (Torres et al., 2017), using default parameters. (ii) Metagenomes with sequence quality scores below 70%, determined using SRA-Tinder^2^ with default parameters, were discarded. (iii) To allow for comparative studies, only metagenomes sequenced using the Illumina sequencing platform and with a minimum of 8 million paired-end reads per library were kept. (iv) Given the CLUE-TERRA consortium’s focus on terrestrial environments, all libraries containing coordinates or terms for sea environments were excluded. After filtering, metagenomes from 1,023 SRA experiments remained from four different studies: (i) “Urban waterways sediment Metagenome” (BioProject: PRJNA267173, SRA: SRP051069) (Saxena et al., 2018), (ii) “New York City MTA subway samples Metagenome” (BioProject: PRJNA271013, SRA: SRP051511) (Afshinnekoo et al., 2015), (iii) “Metagenomics based spatiotemporal study of Chicago River microbiome” (BioProject: PRJNA336577, SRA: SRP080963), and (iv) “Antimicrobial resistance of urban water samples” (BioProject: PRJNA400857, SRA: SRP116665) (Supplementary Table 1). We downloaded the SRA run tables using the SRA run selector^3^ and the SRA accession IDs.

### Pre-processing and library assembly

Metagenome-assembled genomes (MAGs) were recovered using the Multi-Domain Genome Recovery tool (MuDoGeR) (Nunes da Rocha et al., 2022). In short, the raw reads were quality-controlled using metaWrap (Uritskiy et al., 2018) with default parameters. Trimming of raw reads was performed using TrimGalore^4^ with the default settings. High-quality reads (using default Phred scores from TrimGalore) were aligned to potential host genomes using bmtagger (Rotmistrovsky and Agarwala, 2011) using default parameters and the human build 38 patch release 12 database (GRCh38.p12). This alignment aims to remove human DNA contamination and read pairs with only a single aligned read from the metagenomic libraries. We used metaSpades (Nurk et al., 2017) to assemble the reads using default parameters. We then binned the assembled contigs into MAGs using Metabat2 (Kang et al., 2019), Maxbin2 (Wu et al., 2016), and CONCOCT (Alneberg et al., 2014). The recovered bins were refined and dereplicated (Nunes da Rocha et al., 2022). All assembled bins were quality-checked using CheckM (Parks et al., 2015) and filtered for metagenome-assembled genomes (MAGs) based on the following criteria: at least 50% completeness and less than 10% contamination based on CheckM results (medium and high-quality MAGs), and a quality score higher or equal to 50, where quality score = completeness-5*contamination” (Parks et al., 2017; Supplementary Table 2).

### Genomic operational taxonomic unit clustering and taxonomy classification

We used the OTU picking script from Module 5 in the MuDoGeR tool (Nunes da Rocha et al., 2022) with default parameters to cluster the 4,281 MAGs into genomic operational taxonomic units (gOTUs). In short, the tool first filtered out MAGs that did not meet the following criteria: at least 50% completeness, a quality score higher than 50, and an N50 higher or equal to 10,000. It then divided the 2,073 remaining MAGs into taxonomic groups based on their GTDB-tk classifications (Chaumeil et al., 2019). Finally, it used FastANI (Jain et al., 2018) to cluster the MAGs into gOTUs based on an average nucleotide identity (ANI) of 95, which can be used as a proxy for species (Jain et al., 2018). To each gOTU, we assigned a “best bin” that represents the cluster. The “best bin” was defined as the bin with the highest quality score. In the event of a tie, we selected the bin with the lowest number of contigs, then the highest N50, and finally, the lowest strain heterogeneity. GTDB taxon names are often appended with an alphabetic, which means that the taxon is either not monophyletic in the GTDB reference tree or its placement in the tree is unstable (Parks et al., 2022). For simplicity, we have removed these suffixes in our analysis, but all unmodified GTDB-tk classification names are listed in Supplementary Table 3.

### Antimicrobial gene annotation

We used DeepARG-LS (Arango-Argoty et al., 2018) to identify ARGs (ARGs) in each MAG. We first translated the MAG genome sequences to amino acid sequences using the faTrans tool from KentUtils (Kent, 2022). We defined ARGs as present in our MAGs when they met the following DeepARG-LS output criteria: equal or higher than 80% probability, an e-value lower than 1 × 10^−10^, and percent identity of 35% or higher, as done by Wicaksono et al. in a recent study (Wicaksono et al., 2021; Supplementary Table 4). We chose the 35% identity cutoff to utilize the novel ARG prediction power of the DeepARG-LS model. A cutoff of 50% identity or higher allows for the prediction of high-quality ARGs (Arango-Argoty et al., 2018). However, because we are working with MAGs, which largely reflect the uncultured, and thus less known, proportion of urban prokaryotes, it is fitting to allow for the prediction of novel ARGs within our data. In order to test whether our findings resulted from an overestimation of ARGs, we repeated our analyses with the default DeepARG-LS 50% identity cutoff (Supplementary Figures 1–4) We converted all ARG identifiers to upper case to standardize the IDs and removed the differentiation between gene and protein identifiers. The correlation between the numbers of ARGs in an ARG class and the numbers of gOTUs containing at least one MAG with at least one ARG from an ARG class was calculated using Kendall’s rank correlation. Kendall’s tau can be used to evaluate the correlation of non-linear monotonic continuous data as an alternative to Pearson correlation, which assumes linearity, an assumption that our data does not fulfill (Puth et al., 2015).

### Predicting plasmid sequences and virulence factors

We used PlasFlow (Krawczyk et al., 2018) with default parameters and a threshold of 0.7 to predict the sequence types of all sequences that were at least 1,000 base pairs long in all of our MAGs (Supplementary Table 5). Additionally, we aligned the MAG nucleotide sequences to all amino acid sequences belonging to the Victors database of virulence factors (Sayers et al., 2019) using blastx. Virulence factors with an e-value lower than 1 × 10^−10^ and a percent identity above 80 were considered (Supplementary Table 6). The number of virulence factors per gOTU was calculated by counting the unique Victors virulence factors aligned to at least one of the MAGs belonging to the gOTU.

### Prevalence and randomness of ARGs in different taxonomic levels

Prevalences of ARGs and ARG classes in gOTUs were calculated based on the presence and absence of each ARG in each MAG. For each gOTU, the prevalence was calculated as the number of MAGs containing the ARG or ARG belonging to an ARG class, divided by the number of MAGs belonging to the respective cluster, similar to the prevalence definition by Danko et al. (2021) (Supplementary Table 7). Weighted average prevalence (WAP) was calculated per ARG class on the phylum level by multiplying each phylum’s prevalence with the number of gOTU species in that phylum and dividing the result with the total number of gOTUs, and finally adding up all the values: $WAP=\overset{p}{\underset{i=1}{\sum }}\frac{\mathrm{prevalenc}e_{i}\times \mathrm{number}\;\mathrm{of}\;\mathrm{gOTU}s_{i}}{\mathrm{total}\;\mathrm{number}\;\mathrm{of}\;\mathrm{gOTUs}}$, where *p* is the number of unique phyla. For each ARG class, we calculated the normalized mutual information (NMI) per taxonomic class using the taxonomic order level to categorize the data using the “aricode” package in R (Chiquet et al., 2020). In information theory, the mutual information (MI) of two variables is a measure that can compute the mutual dependency between those variables (Press et al., 1992). In other words, this measure quantifies the amount of information obtained about a variable by observing the other random variable. The value of the MI can be between [0, ∞], where a zero value means that there is no dependency between two variables. The regular version of the MI is known as NMI, where values have been normalized to the range [0,1], and bias to the variable with a larger number of bins is removed (Singh et al., 2014). To test if the differences we observed using NMI were significant, we used Pearson’s chi-squared test, which can be used to assess if there is a statistically significant difference between two categorical variables (Xia and Sun, 2017). When the chi-squared test’s significance value was less than 0.05, we assigned a significant difference between the two variables used in the NMI calculation (Supplementary Table 8).

### Phylogenetic tree

Phylogenetic trees were created using the GTDB-tk taxonomic classifications of each MAG or gOTU. The GTDB-tk taxon levels were uploaded to the phyloT (v2) webpage,^5^ (accessed 01.08.2022) to generate the tree (Letunic, 2022). As input, we used the lowest taxonomic level provided by the GTDB-tk classification of each gOTU’s best MAG (Supplementary Table 3). However, not all identifiers were accepted as input in the phyloT tool. In these cases, we first used the phyloT search function to check for an alternate identifier for the same taxonomic level. When that did not result in a valid identifier, we instead used the next higher taxonomic level provided by GTDB-tk classification. At the lowest accepted levels, the 317 gOTUs belonged to 305 GTDB identifiers that were accepted as input into phyloT, out of which 283 are represented as leaves in the final tree. In the tree, gOTUs belonging to the same GTDB identifier cannot be differentiated. The tree and heatmap were plotted using the R package “gtree” (Guangchuang, 2020).

### Data availability statement

Metagenome-assembled genome sequences are available in the NCBI BioProject database (BioProject accession number PRJNA850115). All analyzed data sets are available as Supplementary material on the publisher’s page, and the code is available on GitHub.^6^

## Results

### The recovered urban MAGs are of high quality and taxonomically diverse

**Figure 1:**
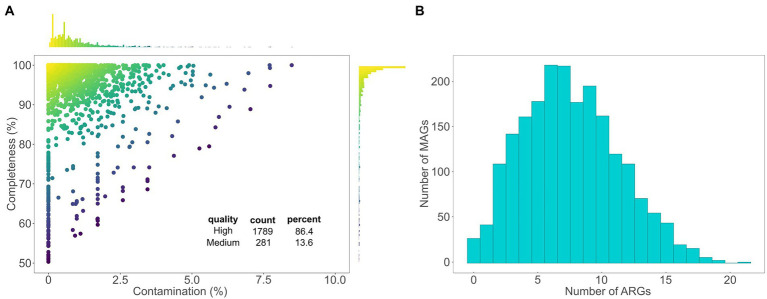
MAG quality and number of ARGs. **(A)** A scatter plot showing the contamination and completeness levels of the 2,073 MAGs that clustered in the gOTU picking. The two histograms show the distribution of contamination and completeness among the MAGs. Each point is colored according to its quality score (Methods). Medium quality MAGs have completeness higher than 50% and contamination lower than 10%. High-quality MAGs have completeness higher than 90% and contamination lower than 5%. All MAGs have a quality score higher than or equal to 50. **(B)** A histogram showing the number of ARGs found in each of the 2,073 MAGs that clustered in the gOTU picking.

We recovered 1,396 medium (completeness >50, contamination <10) and 2,885 high (completeness >90, contamination <5) quality MAGs (4,281 in total) from 1,023 metagenomic samples of four different studies that contained urban terrestrial metagenomes from diverse surfaces (Supplementary Table 1; Supplementary Figure 5). According to the GTDB-tk classification (Chaumeil et al., 2019), the 4,281 recovered MAGs belonged to 17 different phyla, Supplementary Figure 6; Supplementary Table 3. We performed genomic operational taxonomic unit (gOTU) clustering on the 4,281 MAGs. After filtering, 2,073 MAGs were divided into 317 gOTUs (i.e., distinct species; Supplementary Table 3), where each gOTU contains MAGs that belong to the same species. Of the 2,073 MAGs, 13.6% were of medium quality and 86.4% of high-quality (Figure 1). The 317 gOTUs were assigned to 259 known GTDB-tk species and 58 potentially novel species belonging to 13 phyla. The majority (292) of the gOTUs belonged to the four phyla most commonly found in urban metagenomic samples (Danko et al., 2021): *Actinobacteria* (15 gOTUs; 11 known species), *Bacteroidota* (34 gOTUs; 25 known species), *Firmicutes* (53 gOTUs; 26 known species), and *Proteobacteria* (190 gOTUs; 172 known species). Other phyla included *Cyanobacteria* (5 gOTUs; 2 known species) and *Patescibacteria* (12 gOTUs, 1 known species). We identified the presence of ARGs in each MAG using DeepARG-LS (Arango-Argoty et al., 2018). ARGs with equal or higher than 80% probability, an e-value lower than 1 × 10^−10^, and percent identity of 35% or higher (Wicaksono et al., 2021) were considered present. Because we are working with MAGs that largely reflect the uncultured, and thus less known, proportion of urban prokaryotes, it is fitting to allow for the prediction of novel ARGs within our data. Therefore, we chose the 35% percent identity cutoff to utilize the novel ARG prediction power of the DeepARG-LS model. Out of the 2,073 MAGs assigned to gOTUs, 2,046 (98.7%) contained at least one ARG, with an average of 8 ARGs per MAG (Figure 1B).

### Multiple antimicrobial resistance gene classes are highly prevalent in all detected branches of the urban bacterial phylogeny

**Figure 2:**
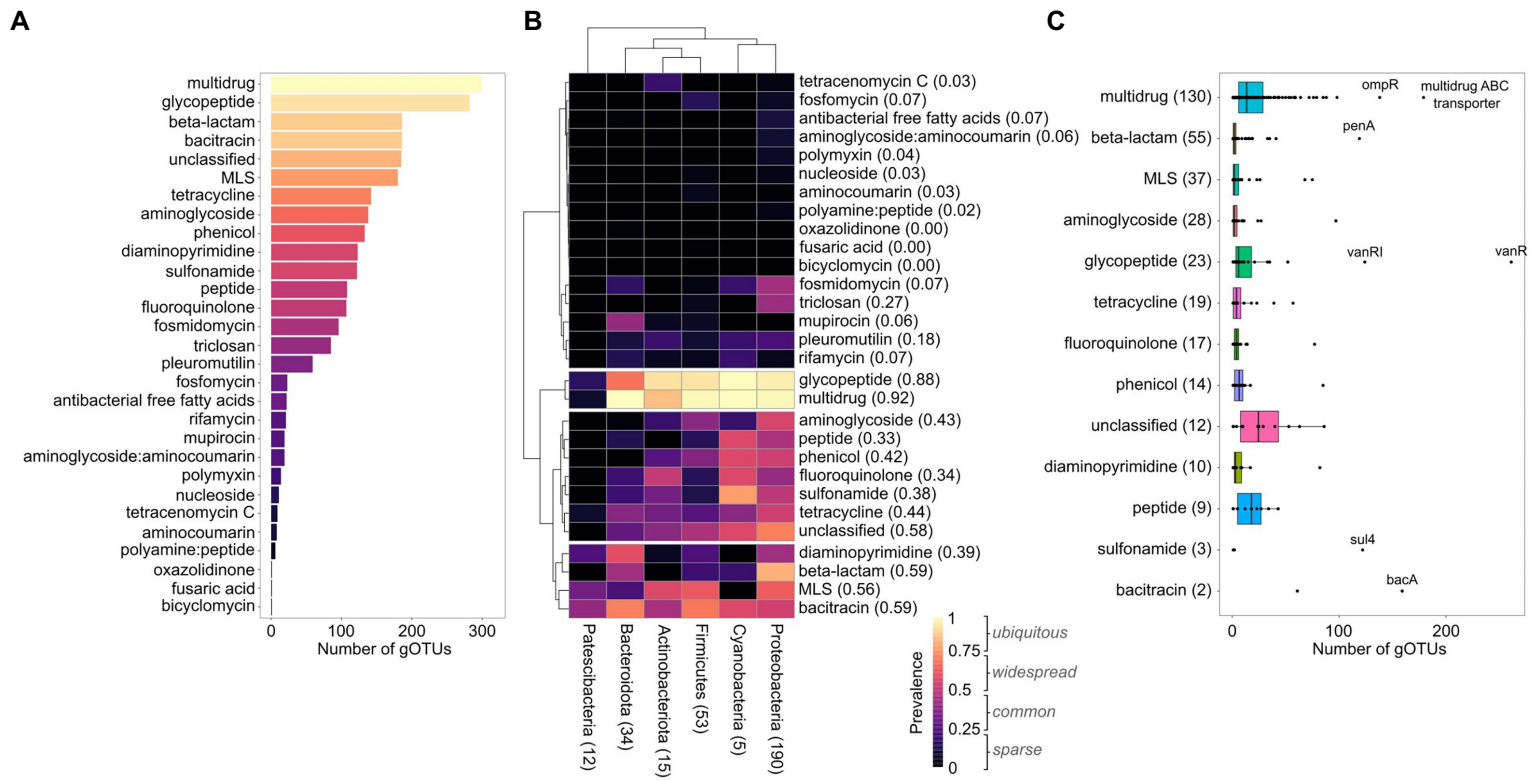
Prevalence of antimicrobial resistance gene classes in urban bacteria. **(A)** A bar plot showing the number of gOTUs that contain ARGs belonging to each ARG class. Colors refer to the prevalence of each ARG class in all gOTUs (i.e., species). **(B)** A heatmap showing the prevalence of ARG classes in the urban taxonomic phylum level. Shown are the number of gOTUs that belong to each phylum. Only phyla with five or more gOTUs are shown. The dendrogram is based on hierarchical clustering with Ward distance between the ARG class prevalence among the four phyla. The weighted average prevalence (WAP) of the ARG class on the phylum level is in parentheses. In gray within the legend, we show the ranges of our defined prevalence categories. **(C)** Boxplots showing the number of gOTUs that contain each ARG within the ARG classes that were present in the ubiquitous, widespread, and common categories from **(B)**. In parentheses are the number of ARGs within the ARG class. ARGs present in at least 1/3 of the gOTUs are labeled.

We found that the ARGs showing resistance to multidrug and glycopeptides had already spread among most urban bacterial gOTUs (Figure 2A). We also saw that ARGs of macrolide-lincosamide-streptogramin (MLS), beta-lactam, and bacitracin were already present in two-thirds of our gOTUs. Beta-lactam, bacitracin, MLS, and unclassified ARGs followed the multidrug and glycopeptide ARGs, being present in over half of all gOTUs (Figure 2A). To investigate the spread of these ARGs in urban bacteria, we calculated the prevalence of all ARG classes among the MAGs within each gOTU (Figure 2B). We defined four categories based on ARG prevalence: i) ubiquitous (prevalence >0.75), ii) widespread (0.5 < prevalence ≤0.75), iii) common (0.25 < prevalence ≤0.5), and iv) sparse (0 ≤ prevalence ≤0.25). Based on the dendrogram in Figure 2B, we observed that, based on their prevalence on the phylum level, the 29 ARG classes could be divided into four groups, each group corresponding to one of the four categories based on the weighted average of the prevalence (WAP) among the four phyla. The first group mainly contained sparse classes, the second group contained the two ubiquitous classes, glycopeptide and multidrug, the third group contained mostly common classes, and the fourth group contained widespread classes.

In total, we found 13 ARG classes that were present in 100 or more gOTUs (Figure 2A). However, we also investigated the prevalence of individual ARGs among the 317 gOTUs and found that 5 out of the 30 ARG classes contained a total of seven individual ARGs that were found in 100 or more gOTUs (Figure 2C). This means that the ARGs within some ARG classes had spread across different gOTUs. In the next section, we dive deeper into the spread of ARGs in the urban prokaryotic tree of life by testing whether ARGs were randomly spread across different taxonomic levels. According to the CARD database, most of the highly prevalent ARGs have target-altering or efflux pump resistance mechanisms (Table 1; Alcock et al., 2019).

**Table 1:** Resistance mechanisms of the ARGs that were found in more than 100 gOTUs.

ARG	ARG class	Resistance mechanism
Multidrug ABC transporter	Multidrug	Antibiotic efflux*
ompR	Multidrug	*Not available in CARD*
penA	Beta-lactam	Antibiotic target alteration
vanR	Glycopeptide	Antibiotic target alteration
vanRI	Glycopeptide	Antibiotic target alteration
sul4	Sulfonamide	Antibiotic target replacement
bacA	Bacitracin	Antibiotic target alteration

Resistance mechanisms were taken from the CARD database (Alcock et al., 2019). *Not available by name in CARD, but because the names include the word “transporter,” we assign the antibiotic efflux mechanism.

Our analysis also showed that the number of ARGs within an ARG class was not indicative of the ARG class prevalence (Figure 2C). The multidrug class was the most prevalent ARG class in our data and had the most individual ARGs. However, *vanR* was the most prevalent ARG in our data and belonged to the glycopeptide class, which encapsulates only a fifth of the number of ARGs in the multidrug class. In addition, the third most prevalent ARG (*bacA*) belongs to the bacitracin class, which contained only two ARGs in our data. To test this observation, we calculated the correlation between the number of unique ARGs per ARG class and its prevalence in our observed gOTUs (Kendall’s tau 0.69). However, this correlation was not observed when looking only at ARG classes belonging to the ubiquitous and widespread categories (Kendall’s tau 0.27).

To test if our results came from an overestimation of ARGs, we repeated our analyses with a percent identity cutoff of 50%, the universal cutoff according to DeepARG (Arango-Argoty et al., 2018), which removed the novel predicted ARGs from the data and allowed us to focus on the high-confidence ARGs. Despite the fewer ARGs (Supplementary Figure 1), we saw a widespread prevalence of the multidrug class and a common spread of the glycopeptide, bacitracin, beta-lactam, MLS, fosmidomycin, and aminoglycoside classes, which was largely driven by the prevalence of these classes among *Proteobacteria* and *Firmicutes* (Supplementary Figures 2, 3). When inspecting the individual high-confidence ARGs, we saw a similar distribution of ARGs among the gOTUs as seen with the results that included the novel predicted ARGs (Figure 2C; Supplementary Figure 4), except for a large reduction in predicted multidrug ABC transporters, which was one of the most abundant ARGs when including novel predicted ARGs. However, the fact that we still saw a widespread prevalence of the multidrug class when removing the novel ARGs indicated that our result did not stem from an overestimation of ARGs.

We found that multidrug and glycopeptide ARGs were highly prevalent in all branches of the urban phylogenetic tree. Several other ARG classes showed similar trends as they were widespread in the urban phylogenetic tree.

### Virulence potential and ARG horizontal gene transfer among urban bacteria

**Figure 3:**
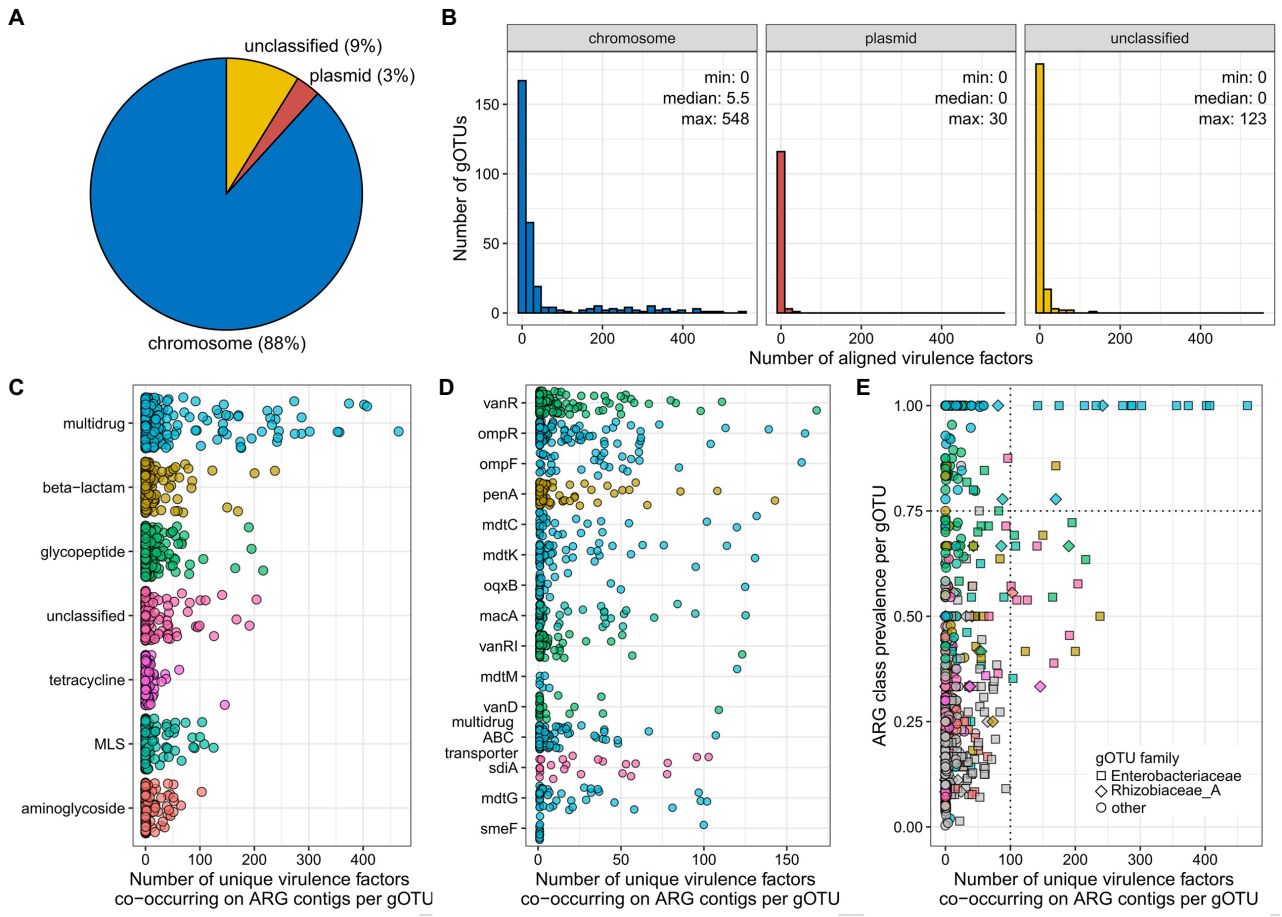
Occurrence of ARGs on plasmids and co-occurrence with virulence factors. **(A)** Percentages of ARGs found on chromosomes, plasmids, or unclassified reads in our data, as determined by PlasFlow (Krawczyk et al., 2018). **(B)** Histograms showing the number of aligned virulence factors (Sayers et al., 2019) per gOTU per read type (chromosome, plasmid, unclassified). **(C)** Number of unique virulence factors that co-occur on contigs with ARGs belonging to each ARG class, per gOTU. Show are only those ARG classes that had more than 100 co-occurring virulence factors in at least one gOTU. **(D)** Number of unique virulence factors that co-occur on contigs with ARGs belonging to the multidrug ARG class, per gOTU. Show are only those ARGs that had more than 100 co-occurring virulence factors in at least one gOTU. **(E)** ARG class prevalence per gOTU as a function of the number of unique virulence factors that co-occur on contigs with ARGs belonging to each ARG class, per gOTU. Highlighted are gOTUs that have both ubiquitous ARG classes and contain more than 100 co-occurring virulence factors for ARGs in the ARG class. The colors correspond to the ARG classes and the shapes to the gOTU taxonomic family. Shown are only those gOTUs that contained more than five MAGs.

In order to estimate the horizontal gene transfer potential of ARGs among urban bacteria, we identified the read type of all contigs in our MAGs as chromosome, plasmid, or unclassified, using PlasFlow (Krawczyk et al., 2018). Our results show that the majority of the urban ARGs in our study were found on chromosomes (91%), and only 2% were found on plasmids (Figure 3A). Additionally, we estimated the virulence of all MAGs by aligning their reads to the Victors database (Sayers et al., 2019), which contains the protein sequences of known bacterial virulence factors. We found that, on average, 41+/−98 (median: 0) virulence factors aligned to each of the gOTUs, ranging from 0 to 550 virulence factors per gOTU (Figure 3B). Interestingly, the ARGs that were found on plasmids did not belong to the highly virulent gOTUs (Figure 3B). However, when we inspect the number of virulence factors per gOTU that co-occur with at least one ARG per ARG class, we saw that the ubiquitous and widespread ARG classes are frequently present in gOTU species that align to more virulence factors (Figure 3C) compared to the other ARG classes (Supplementary Figure 7). We found that gOTUs with ARGs belonging to the multidrug class have a significantly higher number of virulence factors than the other ARG classes (Wilcoxon Rank Sum test, *p*-value range 4 × 10^−14^ to 8 × 10^−3^, Supplementary Table 9). We also found that five of the seven ARGs that were present in at least 100 gOTUs (Figure 2C) were co-occurring with more than 100 virulence factors in multiple gOTUs (Figure 3D). Finally. we observed several gOTUs from the *Enterobacteriaceae* and *Rhizobiaceae* families that contain ARGs from the ubiquitous multidrug or beta-lactam ARG classes in combination with having over 100 virulence factors aligned (Figure 3E; Table 2).

**Table 2:** List of the gOTUs that had more than 100 virulence factors co-occurring with ARGs in ARG classes that were widespread within the gOTU, i.e., ARG class prevalence equal to or higher than 0.75 (Figure 3E).

Family	gOTU	Species	Number of MAGs in gOTU	ARG class	Prevalence	Virulence factors
Enterobacteriaceae	group-160-1	Atlantibacter hermannii	72	Multidrug	1.0	356
Enterobacteriaceae	group-52-1	Atlantibacter sp002358165	6	Multidrug	1.0	175
Enterobacteriaceae	group-164-1	Enterobacter_D sp900116015	13	Multidrug	1.0	374
Enterobacteriaceae	group-20-1	Escherichia coli	11	Multidrug	1.0	465
Enterobacteriaceae	group-255-1	Klebsiella oxytoca	8	Multidrug	1.0	287
Enterobacteriaceae	group-108-1	Klebsiella variicola	7	Multidrug	1.0	273
Enterobacteriaceae	group-108-1	Klebsiella variicola	7	Beta-lactam	0.86	170
Enterobacteriaceae	group-48-1	Kosakonia cowanii	13	Multidrug	1.0	214
Enterobacteriaceae	group-70-1	Leclercia adecarboxylata	156	Multidrug	1.0	407
Enterobacteriaceae	group-133-1	Leclercia adecarboxylata_C	12	Multidrug	1.0	402
Enterobacteriaceae	group-92-1	Leclercia sp002902985	10	Multidrug	1.0	232
Enterobacteriaceae	group-82-1	Mixta calida	12	Multidrug	1.0	142
Enterobacteriaceae	group-65-1	Pseudescherichia sp002918705	11	Multidrug	1.0	302
Enterobacteriaceae	group-125-1	UBA7405 sp000755535	7	Multidrug	1.0	287
Rhizobiaceae_A	group-264-1	Ochrobactrum intermedium	9	Multidrug	0.78	170
Rhizobiaceae_A	group-256-1	Ochrobactrum anthropi	12	Multidrug	1.0	242

### Non-randomly spread ARG classes within the urban phylogenetic tree

**Figure 4:**
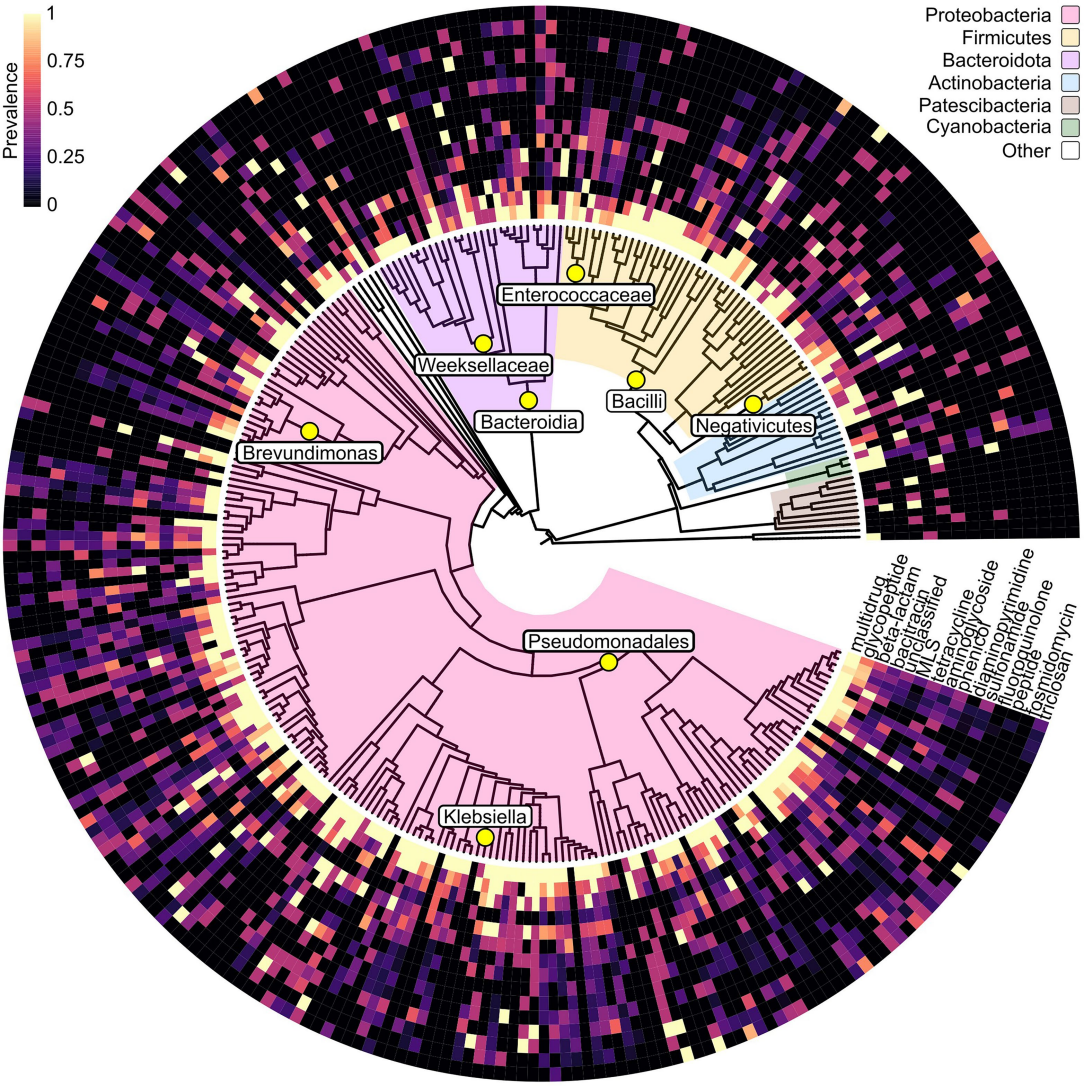
A phylogenetic tree of the urban gOTUs based on their GTDB-tk taxonomy. Tree branches are colored based on their phyla. The surrounding heatmap shows per cluster the prevalence of ARGs belonging to the 15 most prevalent ARG classes in the MAGs belonging to each gOTU. Some gOTUs were identified by GTDB-tk to belong to the same taxonomic category, and the prevalence was then calculated based on all MAGs belonging to all the affected gOTUs. Yellow circles show the presence of non-randomly distributed ARG classes at the taxonomic level displayed in the tree, and the labels show the corresponding taxons. Non-randomness was determined with Normalized Mutual Information (NMI) > = 0.5 and chi-squared *p*-value <0.05. Table 3 lists the taxons with non-random distributions of ARGs within the listed ARG class, along with the NMI and *p*-values.

Seeing the prevalence of ARG classes in urban bacteria, we wanted to test whether the ARGs within these classes were randomly distributed throughout the bacterial taxonomic levels or if there are deterministic events within the phylogenetic tree that affect the distributions of ARGs. We calculated each ARG class’s normalized mutual information (NMI) at every taxonomic level in the phylogenetic tree to assess randomness. We found multiple nodes in the phylogenetic tree where the ARG distributions were flagged as non-random (Figure 4; Table 3). Non-random distributions of ARGs within ARG classes were present in the three most populous phyla; Proteobacteria, Firmicutes, and Bacteroidota. Most ARG classes whose ARGs show non-random distributions within the phylogenetic tree belong to the common and widespread prevalence categories (Figure 2B). The presence of individual ARGs within the taxons flagged as non-random are shown in Supplementary Figure 8.

**Table 3:** Normalized mutual information (NMI) value and chi-squared *p*-value based on the presence of ARGs within each ARG class within the different taxons starred in Figure 4.

Phylum	Taxon	ARG class	NMI	*p*-value	*n*
Firmicutes	Bacilli	Phenicol	0.82	7.7 × 10^−5^	15
Firmicutes	Bacilli	Diaminopyrimidine	0.55	3.5 × 10^−3^	13
Bacteroidota	Bacteroidia	MLS	0.65	8.3 × 10^−3^	11
Bacteroidota	Weeksellaceae	Beta-lactam	0.63	4.6 × 10^−2^	15
Proteobacteria	Negativicutes	MLS	0.52	5.0 × 10^−2^	6
Proteobacteria	Pseudomonadales	Antibacterial_free_fatty_acids	1.00	1.1 × 10^−2^	10
Proteobacteria	Klebsiella	MLS	0.51	4.4 × 10^−2^	8
Proteobacteria	Brevundimonas	Beta-lactam	0.63	4.6 × 10^−2^	12
Proteobacteria	Enterobacteriaceae	Aminoglycoside	0.84	1.4 × 10^−2^	8

Also listed are the taxon phyla and the number of MAGs within the taxon that contained at least one ARG within the ARG class. Here we list only those taxons with an NMI above 0.5 and a *p*-value lower than 0.05. For the complete results, see Supplementary Table 8.

Multiple ARG classes show non-random distributions among the urban bacterial taxonomic levels. It is possible that the spread of these classes is determined by bacterial phylogeny and that they will not reach the high prevalence of multidrug and glycopeptide resistance genes in urban bacteria. When specifically looking at ARGs that were aligned to predicted plasmids, we could not identify any non-random distributions of any ARG classes among the different taxonomic levels (Supplementary Table 10).

## Discussion

Our results show that two ARG classes are highly prevalent among urban bacteria. The taxonomic diversity of the MAGs we recovered and analyzed on a gOTU (i.e., species) level mirrored previously reported urban taxonomic diversity (Danko et al., 2021). In this study, we identified multidrug and glycopeptide ARGs in nearly all urban bacterial species. One reason for the prevalence of the multidrug class is the spread of multidrug ABC transporters (Figure 2C; Table 1), which are among the most common resistance mechanisms in bacteria (Allen et al., 2010) and are commonly found even in pristine environments such as Antarctica (Van Goethem et al., 2018). Additionally, our data showed an alarming ubiquitous prevalence of glycopeptide ARGs and widespread prevalence of MLS, bacitracin, and beta-lactam ARGs. However, despite the high prevalence of these ARG classes in urban bacteria, our analysis suggests that the incidence of horizontal gene transfer among these bacteria is low and that the urban bacteria identified in this study are mostly non-virulent. However, we see that a subset of the putative virulent bacteria contained ARGs that belong to the widespread and ubiquitous ARG classes. However, further studies should be carried out to confirm if the putative virulent bacteria identified in our study are actually virulent. Therefore, we cannot confirm the risk assessment of these bacteria and indicate whether our results are cause for concern.

Our study has some limitations which need addressing. At the time of analysis, the database used to train the DeepARG models had not been updated since April 2020. Therefore, ARGs may be missing in our study. Homology searches for ARGs using the CARD database, updated every three months, are possible but have lower accuracy than deep learning algorithms (Bileschi et al., 2022). Urban microbiomes from similar environments across the globe have different microbial compositions and distributions of ARG classes (Danko et al., 2021). Our samples are largely represented by samples from the United States, and it is thus important to keep in mind that other geographical locations may differ in both taxonomic and ARG diversity and prevalence.

Our results showed that most of the urban bacteria in our study had few virulence factors, which suggests that they are unlikely to be pathogenic to humans. However, we observed many gOTUs with hundreds of virulence factors in addition to highly prevalent ARGs, and the combination of these two factors suggests the presence of antimicrobial-resistant pathogenic bacteria. We have highlighted bacterial species that have high numbers of virulence factors, in combination with the highly prevalent ARG classes, and suggest that further research is needed to assess the potential risk from these species to humans. We identified 15 gOTUs that contained a high number of virulence factors and were ubiquitous in multidrug or beta-lactam ARG classes (Table 2), most of which belonged to the Enterobacteriaceae family, which is known hosts several human pathogens. In addition, some of these identified species and genera are recorded as emerging human pathogens, which further highlights the need for risk assessment of urban microbes (Rodríguez-Medina et al., 2019; Zayet et al., 2021; Jeyaraman et al., 2022).

Most of the ARG classes identified in this study were randomly distributed among the gOTUs (i.e., different species), indicating that there were no deterministic factors on the phylogenetic level that determined the acquiring of ARGs in urban bacteria. This is in line with the results from a recent study where they could not find strong links between phylogeny and ARG diversity in urban sewage (Hendriksen et al., 2019). However, we did see several non-random distributions of ARGs in the urban bacterial phylogenetic tree, which indicates that phylogenetic traits possibly determine the distribution of some ARGs. The question is whether all ARG classes will follow the trends of multidrug and glycopeptide resistance gene spread or if they are determined by phylogeny and will not spread to all urban bacteria. Because antimicrobial resistance in human pathogens is an increasing global problem and that increasing proportions of the global population live in urban environments, antimicrobial resistance genes must not spread fully throughout all urban bacteria. We identified two categories where this is already the case, but we may still be able to prevent other categories from reaching the same level of spreading.

This study includes a large data set of MAGs recovered from over 1,000 urban samples. However, our study covers only a fraction of the possible urban locations worldwide, even with a high number of samples. It would be interesting to perform similar analyses as those we describe in this study on a larger data set (with 10–50 thousand samples) representing different and balanced urban environments. Further research will be required to expand the analysis to more urban metagenomic samples. Another question is whether we see similar antimicrobial resistance gene spreads in other environments (e.g., natural and pristine ecosystems). The urban environment is highly anthropogenic, and it would be interesting to compare our results to further analyses of other highly anthropogenic environments and pristine environments that humans have impacted less.

With this study, we would like to raise awareness that urban ARGs are highly prevalent when analyzing metagenome-assembled genomes and emphasize the need for risk assessment studies at both local and global scales, as these are environments that most of the global population interacts with on a daily basis.

In conclusion, our results showed a high prevalence of antimicrobial resistance genes within the urban bacterial phylogenetic tree. ARGs belonging to two commonly used antimicrobial classes, multidrug and glycopeptide, were ubiquitous among urban bacteria, and multiple other ARG classes follow a similar trend. Additionally, we also saw multiple ARG classes with non-random distributions among urban bacteria. However, we found little evidence that these non-randomly distributed ARGs were transmitted *via* plasmids. Further analyses of additional data sets, more diverse environments with a balanced number of samples, and ARG transmission among bacteria are needed to fully explore the deterministic effects of phylogeny on the distribution of ARGs among urban bacteria.

## Data availability statement

The data availability statement needs to be changed to “Metagenome-assembled genome sequences are available in the NCBI BioProject database (BioProject accession numbers PRJNA843551 and PRJNA850115)".

## Author contributions

SM: investigation, formal analysis, visualization, and writing. JS and AB: methodology and critical review. MS and RT: methodology. JZ: critical review. UR: conceptualization, supervision, and critical review. All authors reviewed and agreed to the content of the manuscript.

## Funding

This work was funded by the NFDI4Microbiota consortium, funded by the Deutsche Forschungsgemeinschaft (DFG, German Research Foundation) – project number 460129525. UR, RT, and JS were financed by the Helmholtz Young Investigator grant VH-NG-1248 Micro “Big Data”.

## Conflict of interest

The authors declare that the research was conducted in the absence of any commercial or financial relationships that could be construed as a potential conflict of interest.

## Publisher’s note

All claims expressed in this article are solely those of the authors and do not necessarily represent those of their affiliated organizations, or those of the publisher, the editors and the reviewers. Any product that may be evaluated in this article, or claim that may be made by its manufacturer, is not guaranteed or endorsed by the publisher.

## Footnotes

^1^ https://www.ufz.de/index.php?en=47300

^2^ https://github.com/NCBI-Hackathons/SRA_Tinder

^3^ https://trace.ncbi.nlm.nih.gov/Traces/study/

^4^ https://github.com/FelixKrueger/TrimGalore

^5^ https://phylot.biobyte.de/

^6^ https://github.com/mdsufz/UrbanARGs

## References

[ref1] Afshinnekoo E. Meydan C. Chowdhury S. Jaroudi D. Boyer C. Bernstein N.. (2015). Geospatial resolution of human and bacterial diversity with City-scale Metagenomics. Cell Syst. 1, 72–87. doi: 10.1016/j.cels.2015.01.001, PMID: 26594662 PMC4651444

[ref2] Alcock B. P. Raphenya A. R. Lau T. T. Y. Tsang K. K. Bouchard M. Edalatmand A.. (2019). CARD 2020: antibiotic resistome surveillance with the comprehensive antibiotic resistance database. Nucleic Acids Res. 48, D517–D525. doi: 10.1093/nar/gkz935, PMID: 31665441 PMC7145624

[ref3] Allen H. K. Donato J. Wang H. H. Cloud-Hansen K. A. Davies J. Handelsman J. (2010). Call of the wild: antibiotic resistance genes in natural environments. Nat. Rev. Microbiol. 8, 251–259. doi: 10.1038/nrmicro2312, PMID: 20190823

[ref4] Alneberg J. Bjarnason B. S. de Bruijn I. Schirmer M. Quick J. Ijaz U. Z.. (2014). Binning metagenomic contigs by coverage and composition. Nat. Methods 11, 1144–1146. doi: 10.1038/nmeth.3103, PMID: 25218180

[ref5] Arango-Argoty G. Garner E. Pruden A. Heath L. S. Vikesland P. Zhang L. (2018). DeepARG: a deep learning approach for predicting antibiotic resistance genes from metagenomic data. Microbiome 6:23. doi: 10.1186/s40168-018-0401-z, PMID: 29391044 PMC5796597

[ref6] Bileschi M. L. Belanger D. Bryant D. H. Sanderson T. Carter B. Sculley D.. (2022). Using deep learning to annotate the protein universe. Nat. Biotechnol. 40, 932–937. doi: 10.1038/s41587-021-01179-w, PMID: 35190689

[ref7] Chaumeil P.-A. Mussig A. J. Hugenholtz P. Parks D. H. (2019). GTDB-Tk: a toolkit to classify genomes with the genome taxonomy database. Bioinformatics 36, 1925–1927. doi: 10.1093/bioinformatics/btz848, PMID: 31730192 PMC7703759

[ref8] Chiquet J. Rigaill G. Sundqvist M. (2020). Aricode: efficient computations of standard clustering comparison measures. Available at: https://CRAN.R-project.org/package=aricode (Accessed August 27, 2022).

[ref9] Corrêa F. B. Saraiva J. P. Stadler P. F. Nunes da Rocha U. (2019). TerrestrialMetagenomeDB: a public repository of curated and standardized metadata for terrestrial metagenomes. Nucleic Acids Res. 48, D626–D632. doi: 10.1093/nar/gkz994, PMID: 31728526 PMC7145636

[ref10] Dancer S. J. Shears P. Platt D. J. (1997). Isolation and characterization of coliforms from glacial ice and water in Canada's high Arctic. J. Appl. Microbiol. 82, 597–609. doi: 10.1111/j.1365-2672.1997.tb03590.x, PMID: 9172401

[ref11] Danko D. Bezdan D. Afshin E. E. Ahsanuddin S. Bhattacharya C. Butler D. J.. (2021). A global metagenomic map of urban microbiomes and antimicrobial resistance. Cells 184:e17, 3376–3393. doi: 10.1016/j.cell.2021.05.002, PMID: 34043940 PMC8238498

[ref12] Guangchuang Y. (2020). Using ggtree to visualize data on tree-like structures. Curr. Protoc. Bioinformatics 69:e96. doi: 10.1002/cpbi.96, PMID: 32162851

[ref13] Hendriksen R. S. Munk P. Njage P. van Bunnik B. McNally L. Lukjancenko O.. (2019). Global monitoring of antimicrobial resistance based on metagenomics analyses of urban sewage. Nat. Commun. 10:1124. doi: 10.1038/s41467-019-08853-3, PMID: 30850636 PMC6408512

[ref14] Jain C. Rodriguez-R L. M. Phillippy A. M. Konstantinidis K. T. Aluru S. (2018). High throughput ANI analysis of 90K prokaryotic genomes reveals clear species boundaries. Nat. Commun. 9:5114. doi: 10.1038/s41467-018-07641-9, PMID: 30504855 PMC6269478

[ref15] Jeyaraman M. Muthu S. Sarangan P. Jeyaraman N. Packkyarathinam R. P. (2022). Ochrobactrum anthropi - An emerging opportunistic pathogen in musculoskeletal disorders - a case report and review of literature. J. Orthop. Case Rep. 12, 85–90. doi: 10.13107/jocr.2022.v12.i03.2730, PMID: 36199934 PMC9499045

[ref16] Kang D. D. Li F. Kirton E. Thomas A. Egan R. An H.. (2019). MetaBAT 2: an adaptive binning algorithm for robust and efficient genome reconstruction from metagenome assemblies. PeerJ 7:e7359. doi: 10.7717/peerj.7359, PMID: 31388474 PMC6662567

[ref17] Kent J. (2022). Genome browser and blat application binaries built for standalone command-line use on various supported Linux and UNIX platforms. Available at: http://hgdownload.soe.ucsc.edu/admin/exe/ (Accessed August 27, 2022).

[ref18] Krawczyk P. S. Lipinski L. Dziembowski A. (2018). PlasFlow: predicting plasmid sequences in metagenomic data using genome signatures. Nucleic Acids Res. 46:e35. doi: 10.1093/nar/gkx1321, PMID: 29346586 PMC5887522

[ref19] Letunic I. (2022). phyloT: phylogenetic tree generator v2. Available at: phylot.biobyte.de (Accessed September 03, 2022).

[ref21] Nunes da Rocha U. Kasmanas J. C. Kallies R. Saraiva J. P. Toscan R. B. Štefanič P.. (2022). MuDoGeR: Multi-domain genome recovery from metagenomes made easy. bioRxiv 496983. doi: 10.1101/2022.06.21.496983 37994269

[ref22] Nurk S. Meleshko D. Korobeynikov A. Pevzner P. A. (2017). metaSPAdes: a new versatile metagenomic assembler. Genome Res. 27, 824–834. doi: 10.1101/gr.213959.116, PMID: 28298430 PMC5411777

[ref23] Parks D. H. Chuvochina M. Rinke C. Mussig A. J. Chaumeil P.-A. Hugenholtz P. (2022). GTDB: an ongoing census of bacterial and archaeal diversity through a phylogenetically consistent, rank normalized and complete genome-based taxonomy. Nucleic Acids Res. 50, D785–D794. doi: 10.1093/nar/gkab776, PMID: 34520557 PMC8728215

[ref24] Parks D. H. Imelfort M. Skennerton C. T. Hugenholtz P. Tyson G. W. (2015). CheckM: assessing the quality of microbial genomes recovered from isolates, single cells, and metagenomes. Genome Res. 25, 1043–1055. doi: 10.1101/gr.186072.114, PMID: 25977477 PMC4484387

[ref25] Parks D. H. Rinke C. Chuvochina M. Chaumeil P.-A. Woodcroft B. J. Evans P. N.. (2017). Recovery of nearly 8,000 metagenome-assembled genomes substantially expands the tree of life. Nat. Microbiol. 2, 1533–1542. doi: 10.1038/s41564-017-0012-7, PMID: 28894102

[ref26] Press W. H. Teukolsky S. A. Vetterling W. T. Flannery B. P. (1992). “Numerical recipes in C” in The Art of Scientific Computing. 2nd. ed. W. H. Press (Cambridge: Cambridge University Press)

[ref27] Puth M.-T. Neuhäuser M. Ruxton G. D. (2015). Effective use of Spearman's and Kendall’s correlation coefficients for association between two measured traits. Anim. Behav. 102, 77–84. doi: 10.1016/j.anbehav.2015.01.010

[ref28] Rodríguez-Medina N. Barrios-Camacho H. Duran-Bedolla J. Garza-Ramos U. (2019). Klebsiella variicola: an emerging pathogen in humans. Emerg. Microb. Infect. 8, 973–988. doi: 10.1080/22221751.2019.1634981, PMID: 31259664 PMC6609320

[ref29] Rotmistrovsky K. Agarwala R. (2011). BMTagger: Best match tagger for removing human reads from metagenomics datasets. Available at: http://www.mmnt.net/db/0/0/ftp.ncbi.nlm.nih.gov/pub/agarwala/bmtagger (Accessed August 11, 2022).

[ref30] Saxena G. Mitra S. Marzinelli E. M. Xie C. Wei T. J. Steinberg P. D.. (2018). Metagenomics reveals the influence of land use and rain on the benthic microbial communities in a tropical urban waterway. mSystems 3, e00136–e00117. doi: 10.1128/mSystems.00136-17, PMID: 29896568 PMC5989131

[ref31] Sayers S. Li L. Ong E. Deng S. Fu G. Lin Y.. (2019). Victors: a web-based knowledge base of virulence factors in human and animal pathogens. Nucleic Acids Res. 47, D693–D700. doi: 10.1093/nar/gky999, PMID: 30365026 PMC6324020

[ref32] Singh B. Kushwaha N. Vyas O. P. (2014). A feature subset selection technique for high dimensional data using symmetric uncertainty. JDAIP 02, 95–105. doi: 10.4236/jdaip.2014.24012

[ref33] Song J. S. Jeon J. H. Lee J. H. Jeong S. H. Jeong B. C. Kim S.-J.. (2005). Molecular characterization of TEM-type beta-lactamases identified in cold-seep sediments of Edison seamount (south of Lihir Island, Papua New Guinea). J. Microbiol. Seoul Korea 43, 172–178. 15880093

[ref34] Torres P. J. Edwards R. A. McNair K. A. (2017). PARTIE: a partition engine to separate metagenomic and amplicon projects in the sequence read archive. Bioinformatics 33, 2389–2391. doi: 10.1093/bioinformatics/btx184, PMID: 28369246 PMC5860118

[ref35] United Nations (2016). Political declaration of the high-level meeting of the general assembly on antimicrobial resistance. United Nations, New York. Available at: https://digitallibrary.un.org/record/842813/files/A_71_L-2-EN.pdf?ln=en (Accessed July 26 2022).

[ref36] United Nations (2018). World economic situation and prospects 2018. United Nations, New York Available at: https://population.un.org/wup/Publications/Files/WUP2018-Report.pdf (Accessed July 26, 2022).

[ref37] Uritskiy G. V. DiRuggiero J. Taylor J. (2018). MetaWRAP—a flexible pipeline for genome-resolved metagenomic data analysis. Microbiome 6:158. doi: 10.1186/s40168-018-0541-1, PMID: 30219103 PMC6138922

[ref38] Van Goethem M. W. Pierneef R. Bezuidt O. K. I. Van De Peer Y. Cowan D. A. Makhalanyane T. P. (2018). A reservoir of ‘historical’ antibiotic resistance genes in remote pristine Antarctic soils. Microbiome 6:40. doi: 10.1186/s40168-018-0424-5, PMID: 29471872 PMC5824556

[ref39] Wicaksono W. A. Kusstatscher P. Erschen S. Reisenhofer-Graber T. Grube M. Cernava T.. (2021). Antimicrobial-specific response from resistance gene carriers studied in a natural, highly diverse microbiome. Microbiome 9:29. doi: 10.1186/s40168-020-00982-y, PMID: 33504360 PMC7841911

[ref40] Wu Y.-W. Simmons B. A. Singer S. W. (2016). MaxBin 2.0: an automated binning algorithm to recover genomes from multiple metagenomic datasets. Bioinformatics 32, 605–607. doi: 10.1093/bioinformatics/btv638, PMID: 26515820

[ref41] Xia Y. Sun J. (2017). Hypothesis testing and statistical analysis of microbiome. Genes Dis. 4, 138–148. doi: 10.1016/j.gendis.2017.06.001, PMID: 30197908 PMC6128532

[ref42] Zayet S. Lang S. Garnier P. Pierron A. Plantin J. Toko L.. (2021). Leclercia adecarboxylata as emerging pathogen in human infections: clinical features and antimicrobial susceptibility testing. Pathogens 10:1399. doi: 10.3390/pathogens10111399, PMID: 34832555 PMC8619052

