# Supplementary material for: Metagenome-assembled genomes indicate that antimicrobial resistance genes are highly prevalent among urban bacteria and multidrug and glycopeptide resistances are ubiquitous in most taxa
Source: Front Microbiol. 2023 Jan 25;14:1037845. doi: 10.3389/fmicb.2023.1037845 (PMC9905122; doi:10.3389/fmicb.2023.1037845)
Supplement: Supplementary file 11 [file Data_Sheet_1.pdf]

# *Supplementary Material*

## 1 Supplementary Figures

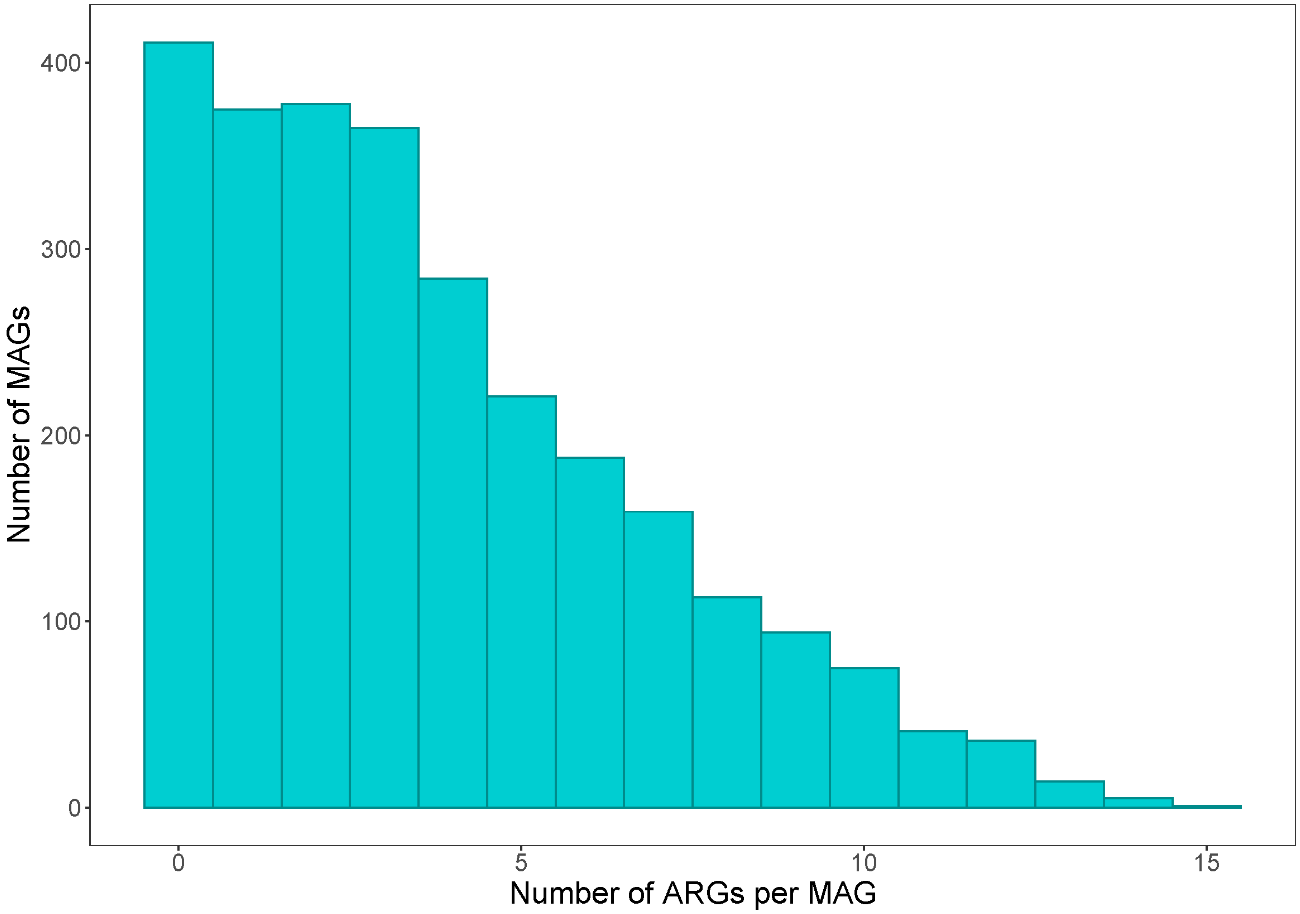


**Supplementary Figure 1**. A histogram showing the ARGs per MAG when using the DeepARG-LS percent identity ≥50%.

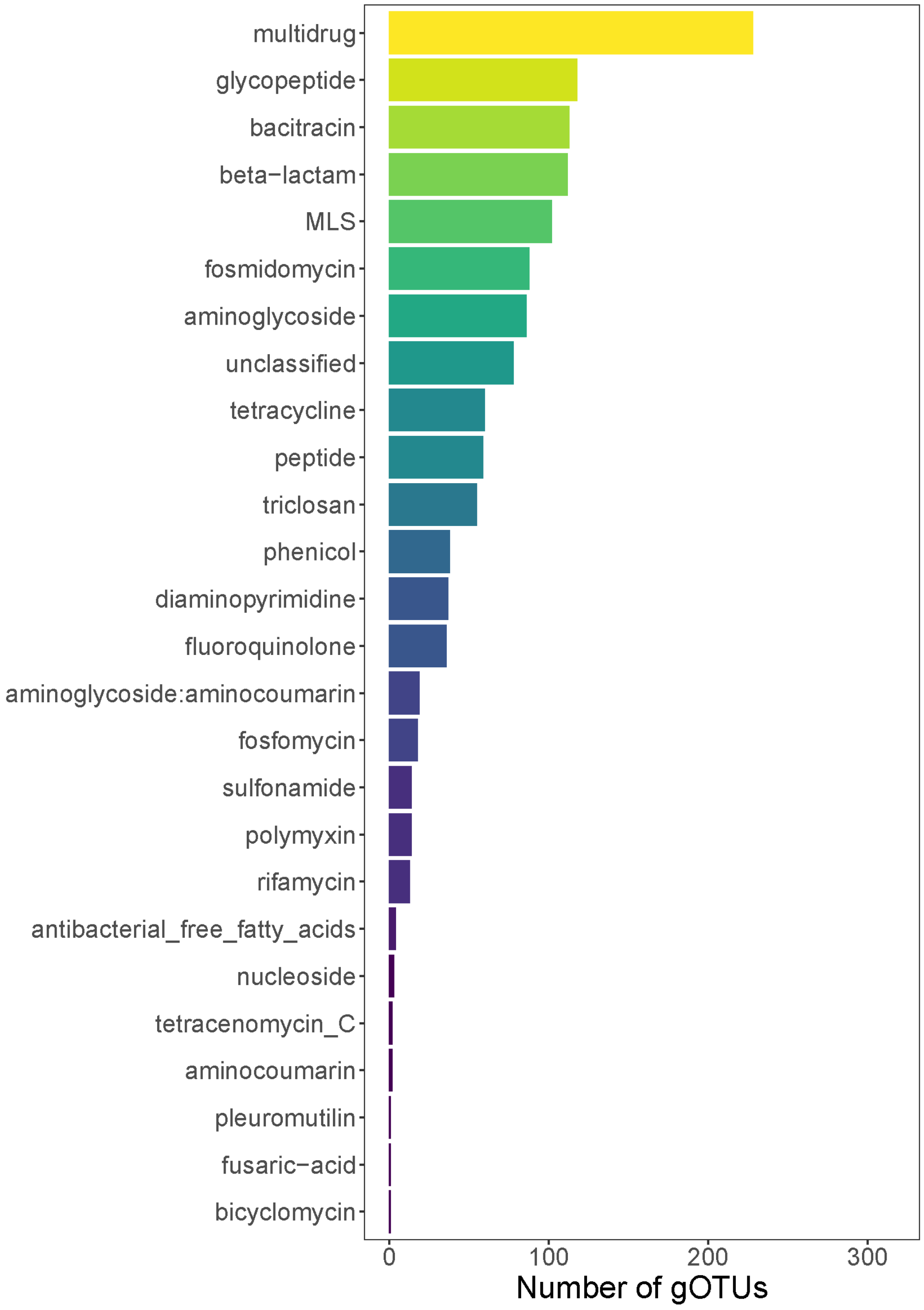


**Supplementary Figure 2**. A bar plot showing the number of gOTUs (i.e., species) that contain ARGs belonging to each ARG class when using the DeepARG-LS percent identity ≥50%.

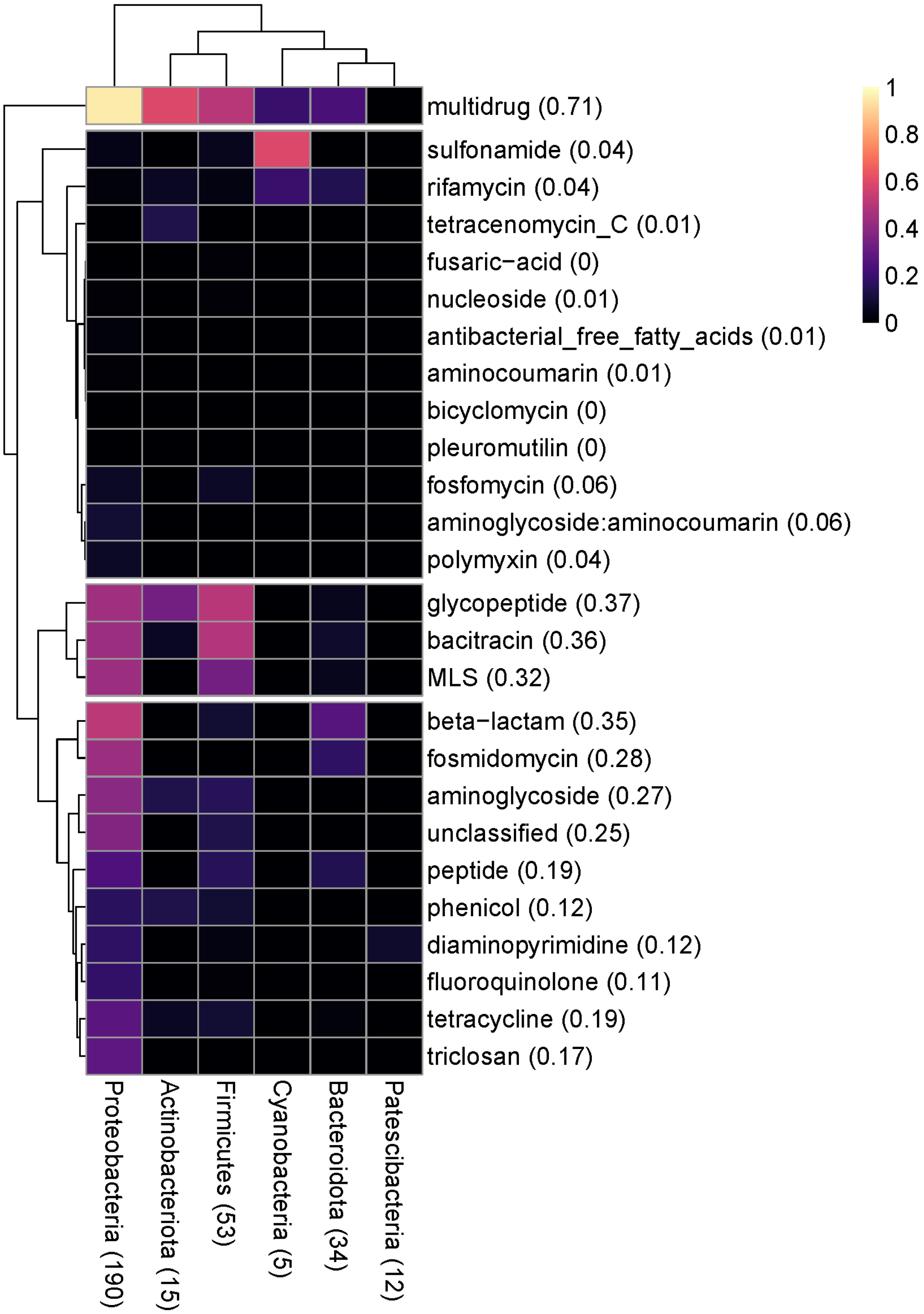


**Supplementary Figure 3.** A heatmap showing the prevalence of ARG classes in the urban taxonomic phylum level when using the DeepARG-LS percent identity ≥50%. Shown are the number of gOTUs that belong to each phylum. Only phyla with five or more gOTUs are shown. The dendrogram is based on Ward distance between the ARG class prevalence among the six phyla. In parentheses is the weighted average prevalence (WAP) of the ARG class on the phylum level.

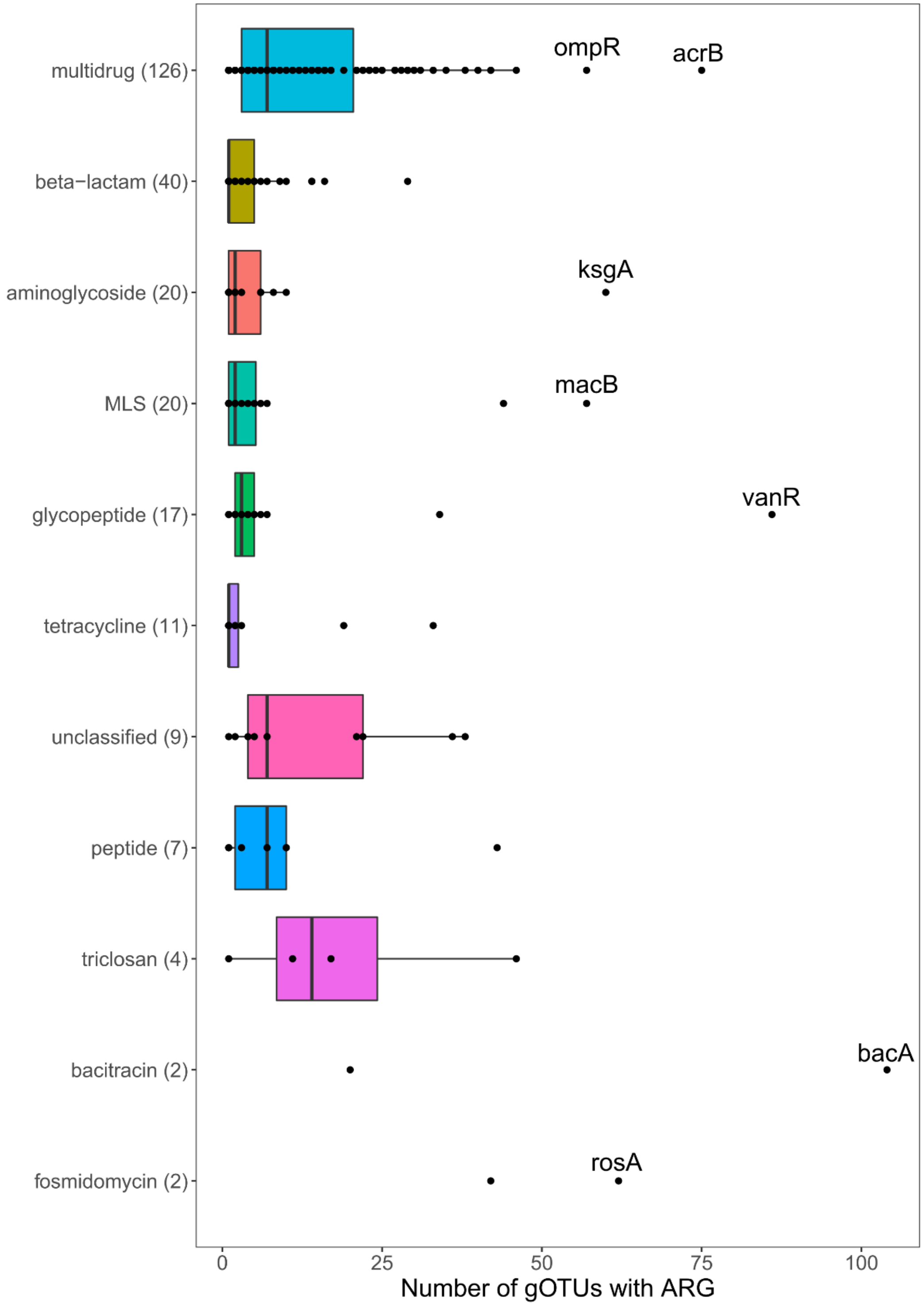


**Supplementary Figure 4.** Boxplots showing the number of gOTUs that contain each ARG within the ARG classes that were present in at least 50 gOTUs when using the DeepARG-LS percent identity ≥50%. In parentheses are the number of ARGs within the ARG class. ARGs present in at least 50 of the gOTUs are labeled.

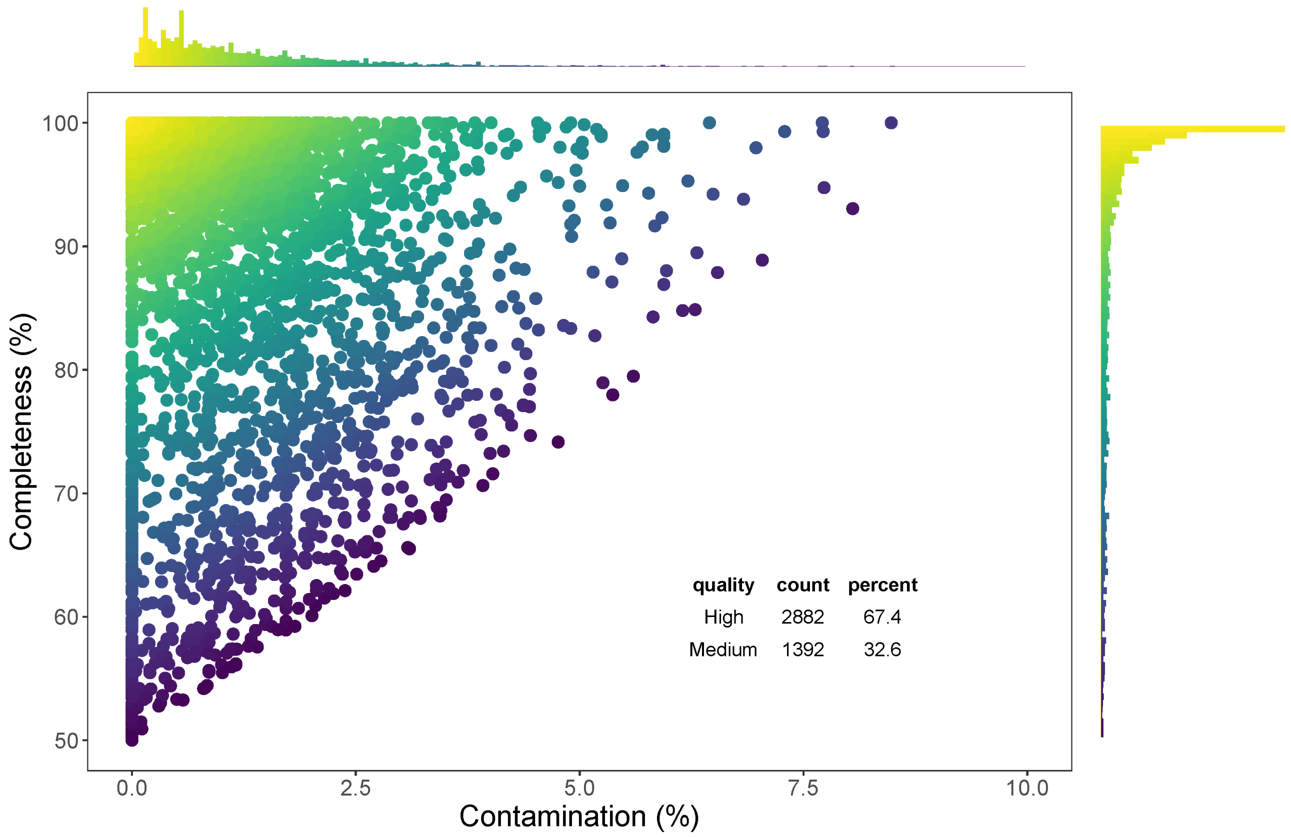


**Supplementary Figure 5.** Completeness and contamination of all 4,274 recovered MAGs. Medium quality: completeness ≥50% and contamination <10%. High quality: Completeness ≥90% and contamination <5%.

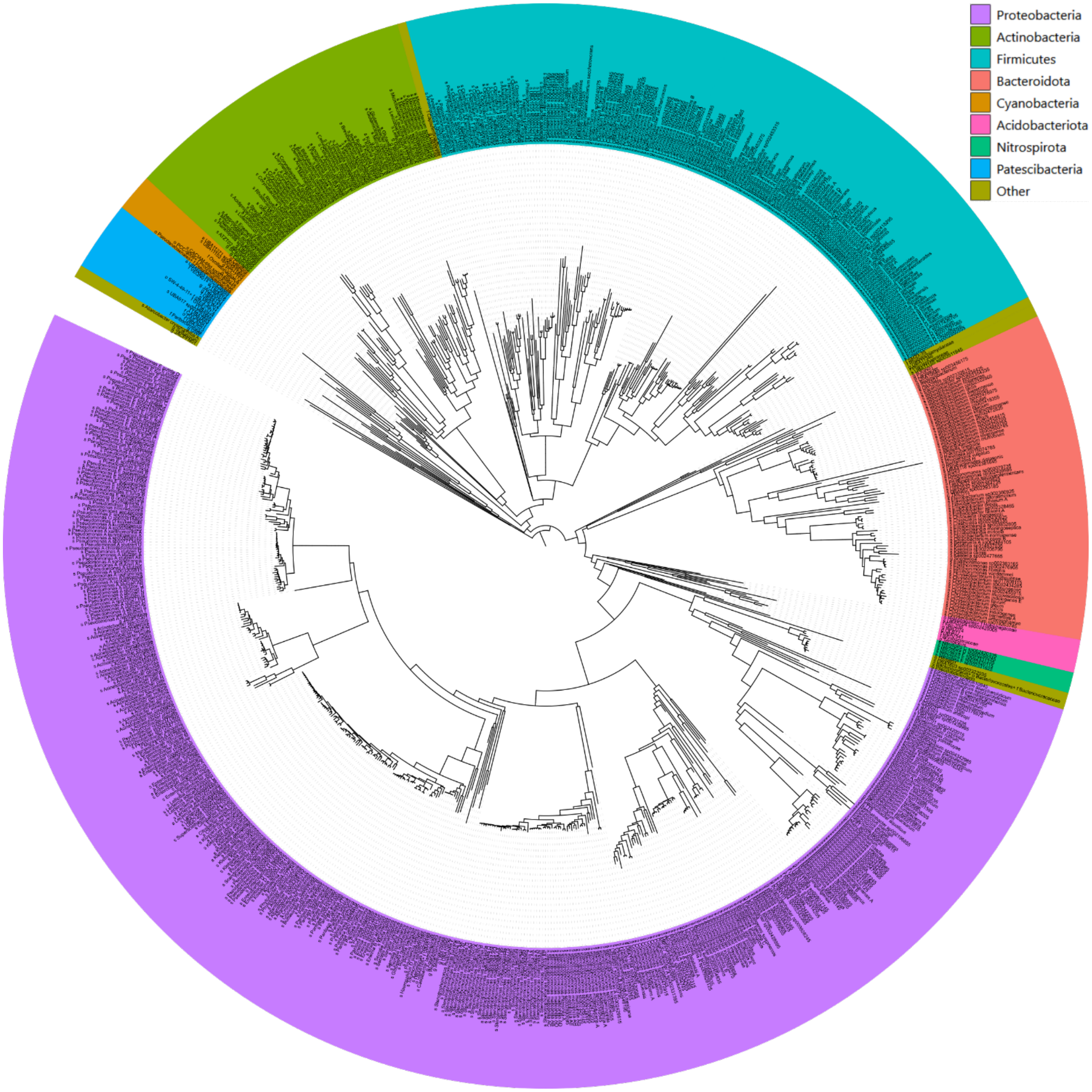


**Supplementary Figure 6.** Phylogenetic tree based on the GTDB identifiers of all 4,274 recovered MAGs. Generated using phyloT (phylot.biobyte.de/) and iTOL (itol.embl.de/). As input, we used the lowest taxonomic level provided by the GTDB-tk classification (Supplementary Table 4). However, not all identifiers were accepted as input in the phyloT tool. In these cases, we first used the phyloT search function to check for an alternate identifier for the same taxonomic level. When that did not result in a valid identifier, we used the next higher taxonomic level provided by GTDB-tk classification instead. The resulting tree contains 801 leaves. The outer circle is colored by phyla.

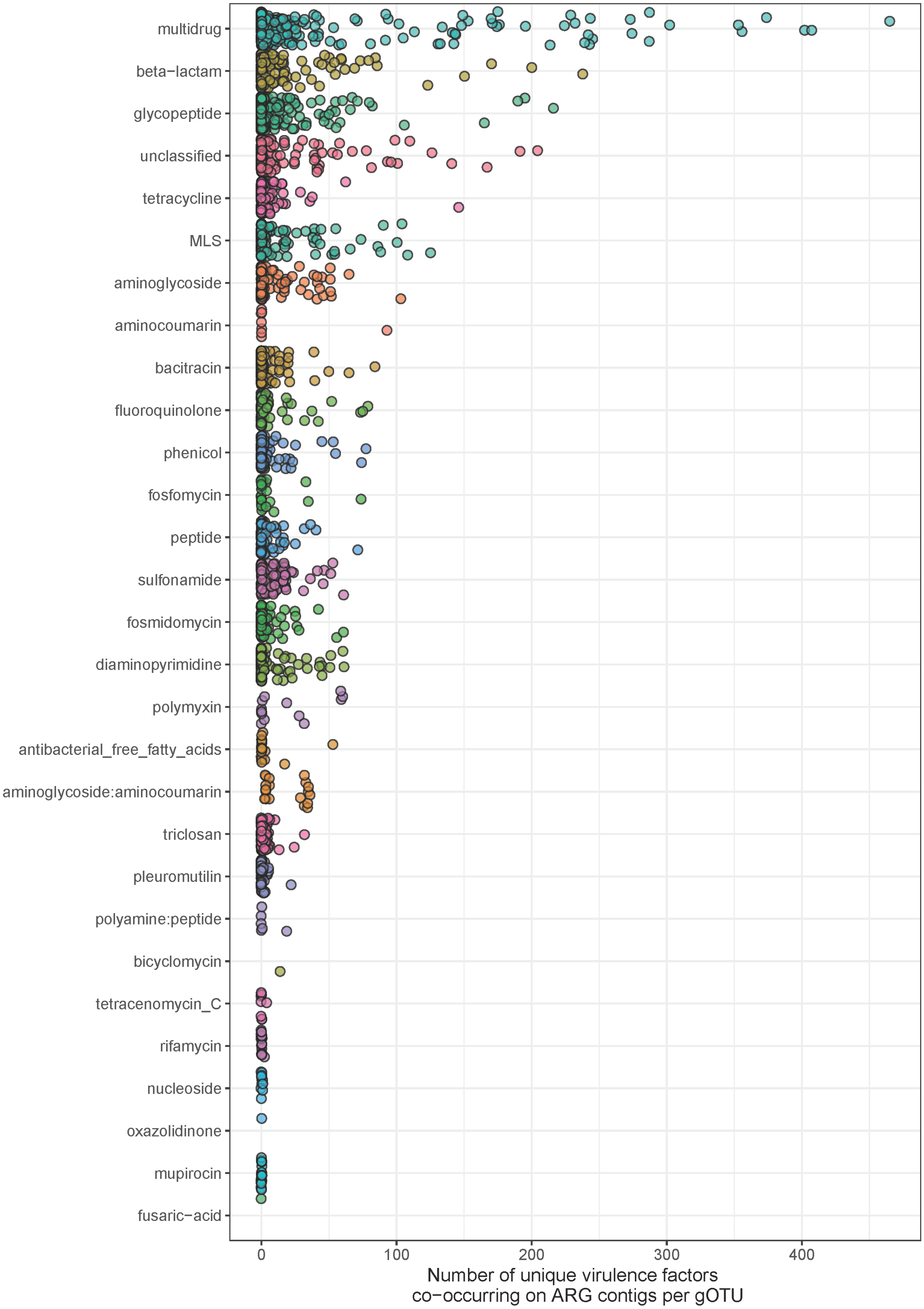


**Supplementary Figure 7.** Number of unique virulence factors that co-occur on contigs with ARGs belonging to each ARG class, per gOTU.

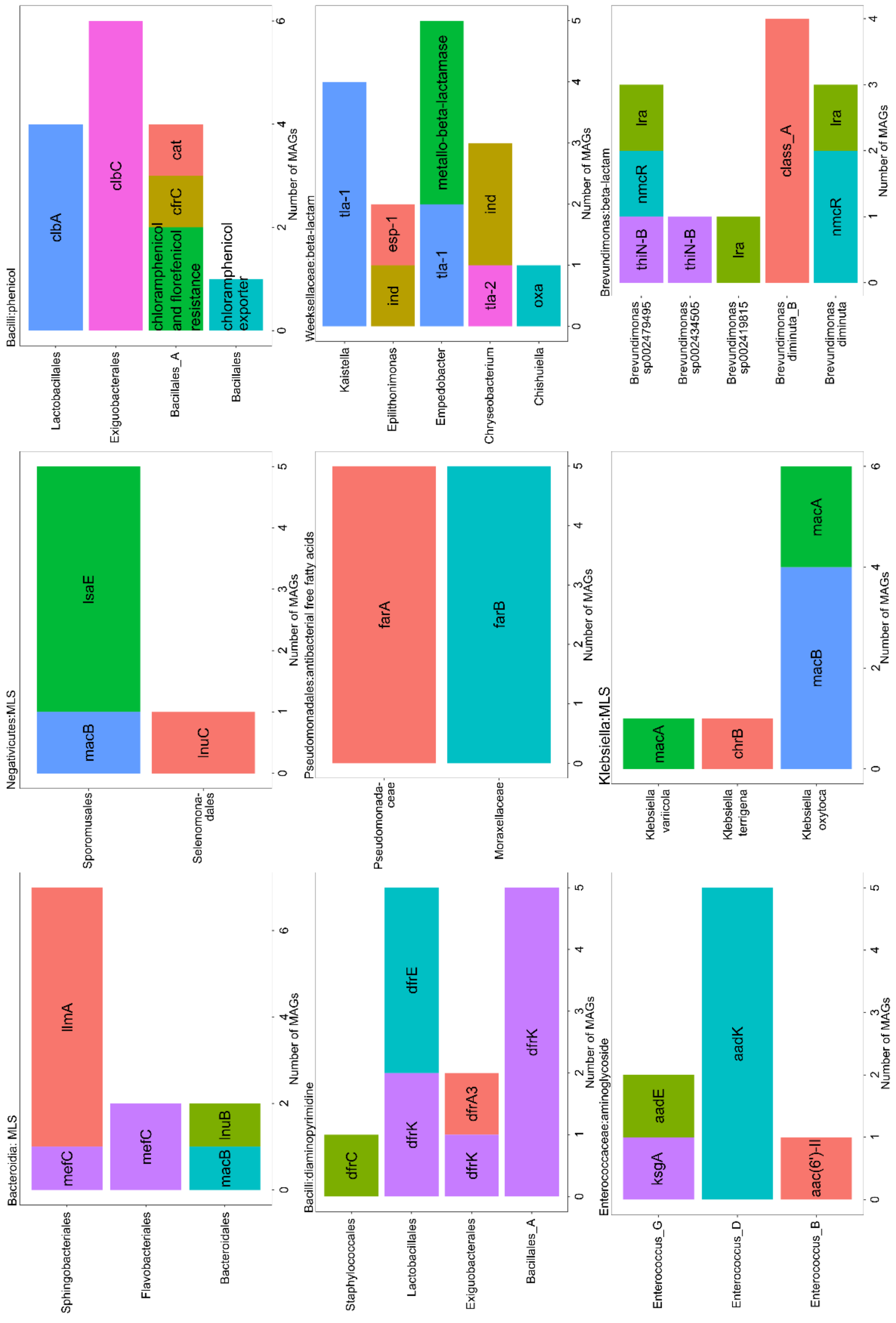


**Supplementary Figure 8.** Number of gOTUs that contain individual ARGs that belong to ARG classes that are non-randomly distributed at different taxon levels (Fig. 3). Each panel (A-Q) show one taxon and one ARG class whose normalized mutual information (NMI) was higher than 0.5 and chi-squared test p-value lower than 0.05. The individual ARGs are labeled.
